# A Plasmonic Mass Spectrometry Approach for Detection of Small Nutrients and Toxins

**DOI:** 10.1007/s40820-018-0204-6

**Published:** 2018-05-17

**Authors:** Shu Wu, Linxi Qian, Lin Huang, Xuming Sun, Haiyang Su, Deepanjali D. Gurav, Mawei Jiang, Wei Cai, Kun Qian

**Affiliations:** 1 School of Biomedical Engineering, Med-X Research Institute, Shanghai Jiao Tong University, Shanghai, 200030 People’s Republic of China; 2 Xinhua Hospital, Shanghai Institute for Pediatric Research, Shanghai Jiao Tong University, Shanghai, 200092 People’s Republic of China

**Keywords:** Plasmonic materials, Laser desorption/ionization, Mass spectrometry, Small nutrients, Toxins

## Abstract

Nutriology relies on advanced analytical tools to study the molecular compositions of food and provide key information on sample quality/safety. Small nutrients detection is challenging due to the high diversity and broad dynamic range of molecules in food samples, and a further issue is to track low abundance toxins. Herein, we developed a novel plasmonic matrix-assisted laser desorption/ionization mass spectrometry (MALDI MS) approach to detect small nutrients and toxins in complex biological emulsion samples. Silver nanoshells (SiO_2_@Ag) with optimized structures were used as matrices and achieved direct analysis of ~ 6 nL of human breast milk without any enrichment or separation. We performed identification and quantitation of small nutrients and toxins with limit-of-detection down to 0.4 pmol (for melamine) and reaction time shortened to minutes, which is superior to the conventional biochemical method currently in use. The developed approach contributes to the near-future application of MALDI MS in a broad field and personalized design of plasmonic materials for real-case bio-analysis.

**Electronic supplementary material:**

The online version of this article (10.1007/s40820-018-0204-6) contains supplementary material, which is available to authorized users.

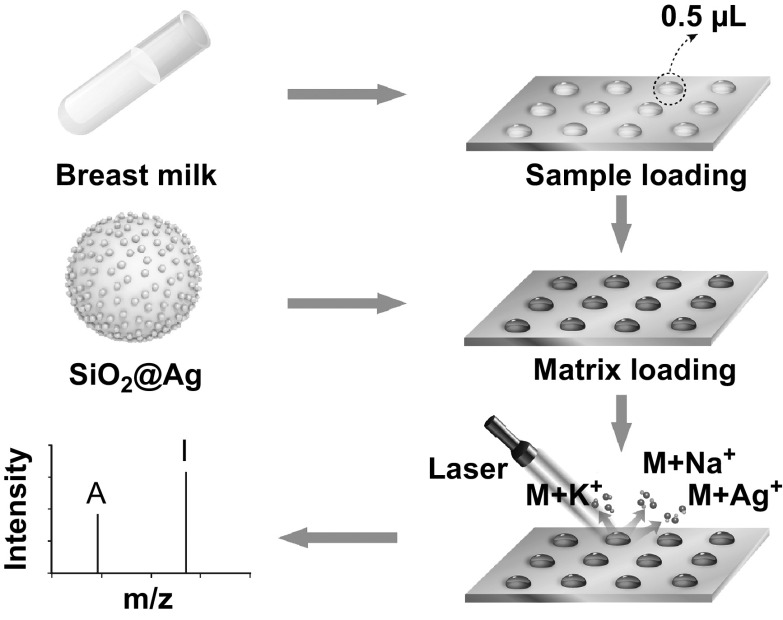

## Highlights

- New materials-based methods. Sensitive detection of small nutrients and toxins (~ pmol) was performed based on plasmonic nanoparticles.
- Advanced analytical performance. Fast quantitation and identification of target molecules (in minutes) were directly achieved in complex emulsion samples.

## Introduction

Nutriology in clinics relies on advanced analytical tools to analyze the molecular compositions of food, which may also provide key information on sample quality and safety [1, 2]. Small nutrient molecules, such as sugar and amino acids, serve as key energy resources and help maintain normal physiological processes in living systems [3–5]. However, detection of small nutrients is challenging, considering the high diversity and broad dynamic range of molecules in food samples. And a further issue is to track small toxins at low concentrations [6–8]. Therefore, a novel tool for analysis of small molecules is in high demand for food industry and clinical nutriology.

Mass spectrometry (MS) affords high accuracy, sensitivity, resolution, and throughput, over conventional approaches, such as nuclear magnetic resonance (NMR), capillary electrophoresis (CE), and biochemical analysis (BCA) [9–13]. Notably, matrix-assisted laser desorption/ionization (MALDI) MS features simple sample preparation and fast analysis for practical use [14–16]. Still, there are two major obstacles for MALDI MS in analysis of small molecules, including (1) selection of tailored matrix materials for specific application with high efficacy; and (2) quantitation of analytes overcoming the non-predictable desorption/ionization behavior [15, 17]. The aforementioned problems need to be addressed before applying MALDI MS-based techniques in biomedical research and industry use.

Plasmonic materials (usually noble metals) enjoy unique photonic, electronic, and thermal properties, due to the surface plasmons with hot carriers [18, 19]. Plasmonic materials facilitate efficient laser desorption/ionization (LDI) processes for MS with designed structural parameters [20–22], which can be advantageous over conventional carbon [23–25] and silicon materials [26–28] suffering from either laser-induced fragmentation or expensive fabrication costs. Compared to the bulk plasmonic matrices, core–shell plasmonic hybrids display higher yields of hot electrons and nanoscale surface roughness, which result in enhanced LDI performance for biomedical use [19, 29]. To date, numerous efforts have been dedicated to the plasmonic MALDI MS worldwide (including our group) [15, 27, 29–31]. A series of core–shell particles have been developed to detect metabolites in diverse solutions, including cerebrospinal fluids [15], serum [29, 30], culturing buffers [32], and exosome lysate [33] recently. Notably, compared to solution analysis as achieved, the application of core–shell plasmonic hybrids remains to be explored for detection of nutrients and toxins toward food industry and clinical nutriology, in a much more complicated colloid system for emulsion analysis.

**Fig. 1:**
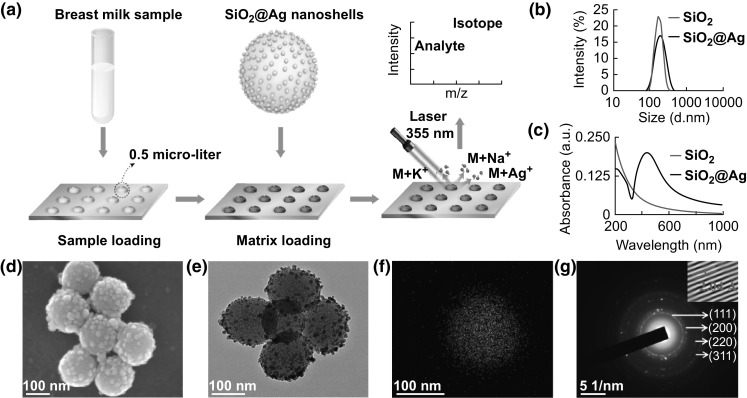
Overall design and characterizations. **a** Schematic diagrams of the experimental workflow. **b** Size distribution of SiO_2_ and SiO_2_@Ag particles by DLS. **c** UV–Vis absorption spectra of SiO_2_ and SiO_2_@Ag particles. **d** SEM, and **e** TEM images of SiO_2_@Ag. **f** Elemental mappings of SiO_2_@Ag (red for Ag and yellow for Si). **g** SAED pattern and HRTEM (inset of g) showing the silver crystal lattice

Herein, we developed a novel plasmonic MALDI MS approach to detect small nutrients and toxins in complex biological emulsion samples (Fig. 1a). We used silver nanoshells (SiO_2_@Ag) with optimized structures as matrices and achieved direct analysis of ~ 6 nL of human breast milk without any enrichment or separation. We performed identification and quantitation of small nutrients and toxins with limit-of-detection down to 0.4 pmol (for melamine) and reaction time shortened to minutes, superior to the conventional biochemical method currently in use. Our approach contributes to the near-future application of MALDI MS in a broad field and personalized design of plasmonic materials for real-case bio-analysis.

## Experimental

### Chemicals and Reagents

Lactose monohydrate (99.0%), melamine (99.0%), ammonium hydroxide (28–30%), tetraethyl orthosilicate (TEOS, 96%), ethanol absolute (99.7%), silver nitrate (99.5%), sodium hydroxide (96%), sodium borohydride (99%), potassium hexacyanoferrate (99.5%), zinc sulfate (99.5%), and Whatman No. 1 filter papers were purchased from Sinopharm Chemical Reagent Beijing Co., Ltd (Beijing, China). [UL-13C6glc]-lactose monohydrate (the isotope contained six ^13^C) was purchased from Cambridge Isotope Laboratories (CIL, USA). Polyvinylpyrrolidone (PVP, MW 40 kDa) and bovine serum albumin (BSA) were purchased from Sigma, USA. All aqueous solutions were prepared using deionized water (18.2 MΩ cm, Milli-Q, Millipore, GmbH) throughout all experiments.

### Materials Synthesis

SiO_2_@Ag core–shell particles were synthesized by coating silver nanoparticles on the surface of silica particles. Firstly, mono-dispersed spherical silica particles were prepared as the core using the well-known Stöber method [34]. Then, the as-prepared silica particles (0.9 g) were dispersed in ethanol absolute (45 mL) to conduct surface coating by silver mirror reaction. Freshly prepared [Ag (NH_3_)_2_]^+^ ion solution (0.59 M, 5 mL) was added to the above particles dispersion and sonicated for 30 min. The dispersed particles were subsequently mixed with 150 mL of PVP ethanol solution (0.5 mM). The suspension was stirred at 70 °C for 7 h for the formation of silver layer. The silver mirror reaction was repeated three times to obtain silver nanoshells [15]. Bare silver nanoparticles were prepared as reported [35]. Briefly, NaBH_4_ solutions ranging in concentration from ~ 5.0 × 10^−4^ to 2.0 × 10^−3^ M were chilled in an ice bath under vigorous stirring to form Ag nanoparticles with different sizes. Then, AgNO_3_ (10 mL, 1.0 × 10^−3^ M) was added and the solution turned light yellow after reaction. The products were washed with ethanol and deionized water three times and dried at 60 °C and then stored as powders.

### Materials Characterization

Transmission electron microscopy (TEM), selected area electron diffraction (SAED) pattern, high-resolution transmission electron microscopy (HRTEM), scanning transmission electron microscopy (STEM), and elemental mapping images were obtained using a JEM-2100F transmission electron microscope (JEOL, Japan). During experiments, ethanol suspension of materials (~ 8–10 μL) was deposited onto a copper grid before observation. Scanning electron microscopy (SEM) images and energy-dispersive X-ray (EDX) spectra were recorded on an S-4800 field emission scanning electron microscope (Hitachi, Japan) by dropping the material suspensions on aluminum foil. Quantitative elemental analysis was performed on an iCAP6300 inductively coupled plasma optical emission spectrometer (Thermo Fisher Scientific, USA). Room temperature optical absorption spectra of the materials were collected on a UV1900 UV–Vis spectrophotometer (AuCy, China). Zeta potential and dynamic light scattering size measurements were taken on a Nano-ZS90 instrument in water at 25 °C (Malvern, Worcestershire, UK).

### Bio-samples Preparation

Standard molecules (e.g., lactose and melamine) were dissolved in deionized water by step-wise dilutions with concentrations ranging from 1 μg μL^−1^ to 0.1 ng μL^−1^, to study the limit-of-detection (LOD). Standard lactose was mixed with salt (NaCl, 0.5 M) and the bovine serum albumin (BSA, 5 mg mL^−1^) to explore detection efficiency in complex samples. Raw cow milk was spiked with melamine at a concentration of 0.5 ng μL^−1^. Human breast milk samples on 42 days postpartum were collected from 20 volunteers. The samples were kept in sterile tubes and stored at − 80 °C until use. Before sample collection, a written informed consent was given to each volunteer. The investigation protocol in this study was approved by the Ethical Committee of Xinhua Hospital (XHEC-C2014016) and School of Biomedical Engineering, Shanghai Jiao Tong University.

### MALDI MS Analysis

Silver nanoshells were dispersed in deionized water at a concentration of 0.5 mg mL^−1^ for matrix use. In a typical MALDI MS experiment, 0.5 μL of analyte solution was spotted on the stainless steel target plate and dried in air at room temperature, followed by adding 0.5 μL of matrix slurry which was dried before MALDI MS analysis. Mass spectra were recorded on a 5800 Proteomics Analyzer (Applied Biosystems, Framingham, MA, USA) equipped with the Nd:YAG laser (1 kHz 355 nm). Spectra acquisitions were carried out in the positive reflector ion mode, employing delayed extraction with a repetition rate of 200 Hz and an acceleration voltage of 20 kV. The delay time for this experiment was optimized to 200 ns. The number of laser shots was 200 per analysis for all LDI MS experiments. The *m*/*z* range was chosen according to the mass of the sample. The tandem mass spectra (MS/MS) of selected peaks of lactose and melamine from bio-samples were collected and compared with standards for identification purpose. MS/MS were performed with the precursor mass ± 0.50 Da. All spectra were directly used without any smoothing procedures [15].

### Molecules Quantification

For isotopic quantification, standard lactose was dissolved in deionized water by step-wise dilutions to obtain the concentrations ranging from 50 to 800 ng μL^−1^. The lactose isotope was dissolved in deionized water at a concentration of 100 ng μL^−1^. Then, the standard lactose solutions were mixed with lactose isotope solution at a volume ratio of 1:1 to obtain the calibration curve. Typically, lactose quantitation was performed by considering the area under the peaks of sodium or potassium adducts of the analyte and internal standard. The relative peak area ratio of the analytes/isotopes (A/I) from five independent experiments was recorded with data shown as the mean ± SD (*n* = 5) for intra-batch analysis. Five replicates were detected for batch-to-batch analysis. To further validate the isotopic quantification method, intraday results were collected by analyzing the 40- and 80-μg μL^−1^ standard lactose solutions. Interday results were collected by analyzing the same samples over five consecutive days to calculate the coefficient of variation (CV).

For biochemical quantitation, the commercial lactose and d-galactose assay kit was purchased from Megazyme International, Ireland. In brief, the enzymatic method required sample purification using the Carrez method [36, 37]. Breast milk (100 μL) was added into a 10-mL volumetric flask with 6 mL of deionized water and mixed at 50 °C for 15 min. Then, 200 μL of Carrez I solution (85 mM potassium hexacyanoferrate) was added, followed by 2 mL of Carrez II solution (250 mM zinc sulfate). Next, 400 μL of 100 mM NaOH was added and the mixture was diluted to a volume of 10 mL with deionized water. Finally, after filtration through Whatman No. 1 filter paper, the detection assay was performed using the AuCy UV1900 spectrophotometer.

## Results and Discussion

### Preparation and Characterization of Materials

Silver nanoshells were synthesized by silver mirror reactions, through the multi-cycled coating of silver nanoparticles on the surface of silica spheres. We used mono-dispersed silica spheres (by the Stöber method) with an average size of 167.1 ± 1.3 nm and polydispersity index (PDI) of 0.024 ± 0.010 (Fig. S1 and Table S1), according to dynamic light scattering (DLS) analysis. Three cycles of silver mirror reactions were conducted for material optimization, and the resulting SiO_2_@Ag had an increased size of ~ 180 nm (Fig. 1b), owing to the introduction of the silver layer. In addition, silver nanoshells were negatively charged with a zeta potential of − 21.0 ± 0.8 mV (Table S1), which was ideal for the production of Na^+^/K^+^/Ag^+^ adducts during LDI. We also prepared bare silver nanoparticles with different sizes of 14.5 ± 1.3, 34.3 ± 2.5, and 94.3 ± 7.7 nm for control experiments (Fig. S2 and Table S1). Notably, the silver nanoshells displayed distinct optical properties from the silica spheres (Fig. 1c), with an absorption peak at ~ 433 nm by ultraviolet–visible (UV–Vis) spectroscopy owing to the Mie plasmon resonance excitation from silver nanoparticles [38]. Therefore, we demonstrated that the silica-silver core-shell particles with absorption band close to 355 nm (the wavelength for Nd:YAG laser used for LDI subsequently) was ideal for matrix use in MS.

The structure of silver nanoshells was further investigated by electron microscopy methods. As shown in the SEM image (Fig. 1d), the silica spheres were loaded with silver nanoparticles layer (~ 10 nm) to form specific nano-gaps on surface, agreed to TEM image (Fig. 1e) and STEM (for elemental mapping) images (Figs. 1f and S3a–c). A clear SAED pattern was observed, confirming polycrystalline silver with [111], [200], [220], and [311] rings as indexed (Fig. 1g) [39]. In addition, HRTEM displayed the typical inter planar spacing of 2.04 Å for silver composites along the [200] direction (inset of Fig. 1g) [39–41]. In order to check the composition of the materials, we recorded the corresponding EDX spectra to demonstrate the presence of silver (Fig. S3d) and further quantitated the silver loading ratio of 14.87 wt% through elemental analysis. The above results were consistent with previous DLS and UV–Vis analyses, validating the structures of silver nanoshells. Nano-gaps of silver nanoshells afforded nanoscale roughness and crevices, for selective trapping of small molecules (MW ~ < 500 Da) for energy transfer, rather than large molecules [15, 32, 33].

### Detection and Identification of Small Nutrients

**Fig. 2:**
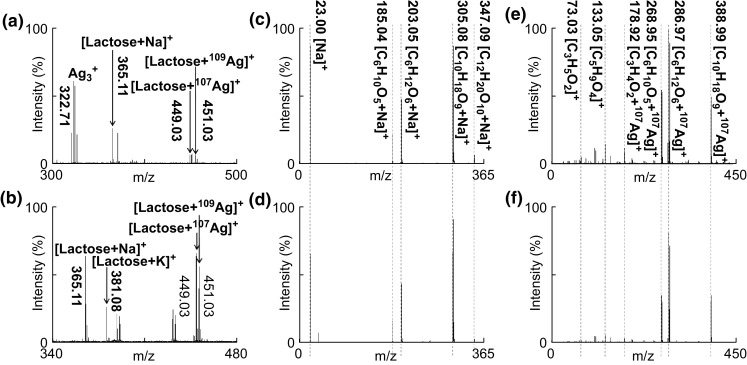
MALDI MS analysis. **a** Mass spectrum of 50 ng μL^−1^ lactose in a 5-mg mL^−1^ bovine serum albumin solution containing 0.5 M NaCl. **b** Mass spectrum of 6.25 nL of breast milk sample with 80-fold dilution. Tandem mass spectra of sodium adducted lactose at an *m*/*z* of 365.11 for [M + Na]^+^ in **c** standard sample and **d** breast milk. Tandem mass spectra of silver adducted lactose at an *m*/*z* of 449.03 for [M + ^107^Ag]^+^ in **e** standard sample and **f** breast milk

The silver nanoshells were used for direct LDI MS detection of small nutrients, using lactose as the standard. Considering the existence of salts and proteins in real-case samples, we studied the salt tolerance and protein endurance of silver nanoshells. We obtained sodium adducted signal at an *m*/*z* of 365.11 [M + Na]^+^ and the silver adducted signal at an *m*/*z* of 449.03 [M + ^107^Ag]^+^ and 451.03 [M + ^109^Ag]^+^ in the mass spectrum (Fig. 2a), dealing with a bio-mixture containing proteins (BSA, 5 mg mL^−1^) and salts (NaCl, 0.5 M) with trace lactose. We also demonstrated the better performance of silver nanoshells in LDI MS analysis of small molecules (lactose and melamine), over bare silver nanoparticles with different sizes and silica spheres (Fig. S4). Further, trace breast milk sample (down to 6.25 nL, Fig. S5) was detected. We observed a series of molecular peaks in the low mass range (~ < 500 Da, Fig. 2b), without any apparent signals in high mass range (Fig. S6). Competitive silver ions may cationize molecules containing polar functional groups (e.g., –OH) via an ion–dipole interaction [42, 43] and π-bonds as per the Dewar model [44, 45]. Due to the selective LDI process being similar to previous reports, we concluded that the silver nanoshells enabled detection of small nutrients (e.g., lactose) in trace complex bio-mixtures without any separation or enrichment.

An MS/MS analysis of the molecular peaks of lactose was performed for identification purpose. The molecular peaks were fragmented at an *m*/*z* of 365.11 for [M + Na]^+^ (standards and milk samples in Fig. 2c, d), providing fragments ions at an *m*/*z* of 185.04 for [C_6_H_10_O_5_ + Na]^+^, 203.05 for [C_6_H_12_O_6_ + Na]^+^, 305.08 for [C_10_H_18_O_9_ + Na]^+^, and 347.09 for [C_12_H_20_O_10_ + Na]^+^. Similarly, we fragmented the molecular peaks at an *m*/*z* of 449.03 [M + ^107^Ag]^+^ (standards and milk samples in Fig. 2e, f), providing fragments ions at an *m*/*z* of 133.05 for [C_5_H_9_O_4_]^+^, 178.92 for [C_3_H_4_O_2_ + ^107^Ag]^+^, 286.97 for [C_6_H_12_O_6_ + ^107^Ag]^+^, and 388.99 for [C_10_H_18_O_9_ + ^107^Ag]^+^. By comparing these MS/MS results, we identified the lactose in breast milk in situ. Besides the accuracy of MS (~ ppm), the presence of multi-signals for Na^+^/K^+^/Ag^+^ adduction increased the confidence levels of identification. Moreover, MS/MS analysis confirmed the structure of the small molecules and displayed strong application potential when coupled with silver nanoshells.

### Isotopic Quantification in Milk Samples

**Fig. 3:**
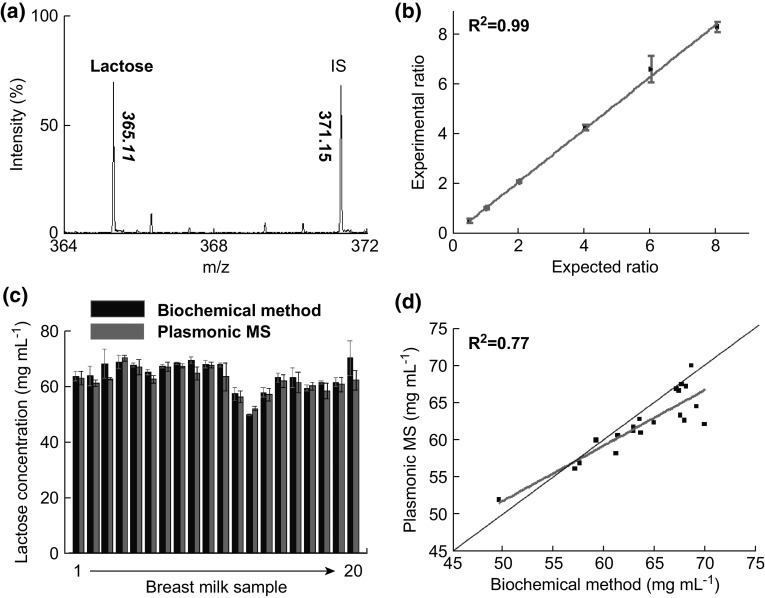
Quantitation of small nutrients. **a** Representative mass spectrum of lactose with its isotope as the internal standard (IS). **b** The calibration curve obtained by plotting the experimental ratio of analyte/isotope (A/I) for sodium adducts as a function of the expected ratio of A/I for lactose. **c** Comparison of results obtained by the plasmonic MS and biochemical method for 20 breast milk samples. **d** Linear correlation of the quantification results from the plasmonic MS and biochemical method

LDI MS is normally qualitative or semiquantitative, due to the non-predictable ionization process [17, 46, 47]. For quantification, we spiked isotopes as the internal standards (IS), which afforded molecular peaks at an *m*/*z* of 371.11 [M + Na]^+^ for the isotopes using the silver nanoshells (Fig. 3a, one representative mass spectrum of an analyte from five experiments; the other four spectra can be found in Fig. S7). The calibration curves for the sodium (Fig. 3b) and silver adducts (Fig. S8) were recorded with the analyte/IS ratios ranging from 0.5 to 8. Potassium adducts were not used due to low peak intensities. We calculated the coefficient of determination (*R*^2^) of 0.99 for both the sodium and silver adducts, demonstrating isotopic quantification successfully coupled with the silver nanoshells-assisted LDI MS. We validated the quantification method for lactose by intraday, interday, intra-batch, and batch-to-batch analysis as summarized in Table S2.

The lactose levels in 20 breast milk samples were quantitated by the plasmonic MS and compared to the conventional biochemical method (Fig. 3c, Table S3). The isotopic quantification afforded an average recovery of 105.3% and a coefficient of variation (CV) of 6.2%, which was comparable to the enzymatic kit that afforded an average recovery of 105.0% and a CV of 3.6% (Fig. S9). These two approaches showed reasonable consistency as per the linear fit method, with an *R*^2^ of 0.77 (Fig. 3d). The biochemical method is the current gold standard in use, relying on the reaction between analytes and enzymes for colorimetric detection to quantitate small molecules [48]. More specifically, the biochemical method is indirect in nature, detecting galactose based on the hydrolysis of lactose into galactose and glucose by *β*-galactosidase. In contrast, the plasmonic MS method directly detects lactose. Notably, plasmonic MS can be advantageous in terms of speed (reaction free, in minute overall analysis) and sensitivity (~ 6 nL of sample required), compared to the biochemical method that requires several hours for analysis (including bio-reactions and pre-treatment steps) and utilizes milliliters of sample.

### Sensitive Detection of Small Toxins

**Fig. 4:**
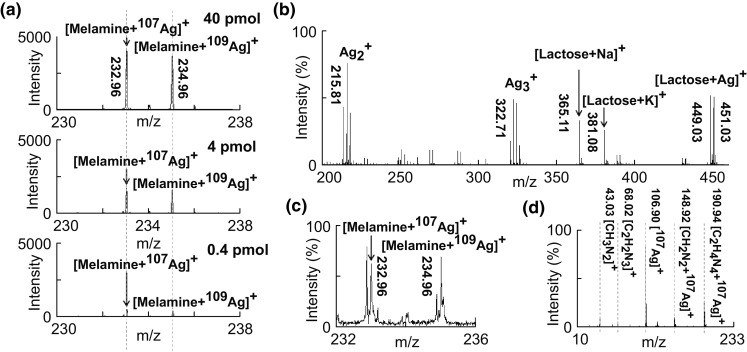
Detection of toxins. **a** Plasmonic MS analysis of melamine prepared in a concentration gradient. Plasmonic MS analysis of raw milk samples spiked with melamine at a concentration of 0.5 ng μL^−1^, showing **b** an *m*/*z* range from 200 to 450, **c** an *m*/*z* range from 232 to 236, and **d** the tandem mass spectrum of melamine at an *m/z* of 232.96 for [M + ^107^Ag]^+^

Besides the nutrient molecules, our approach achieved the detection of small toxins as well. For example, we detected standard melamine solutions prepared in gradient concentrations, affording molecular peaks at an *m*/*z* of 232.96 [M + ^107^Ag]^+^ and 234.96 [M + ^109^Ag]^+^ (Fig. 4a). The experimentally determined LOD of melamine was below 0.1 ng μL^−1^ (*S*/*N* > 3) and was comparable to the best results of previously reported methods [49–51]. A melamine-spiked milk sample was directly detected without any separation or enrichment (Fig. 4b), similar to the detection of lactose, showing an *m*/*z* of 232.96 for [M + ^107^Ag]^+^ and 234.96 for [M + ^109^Ag]^+^ (Fig. 4c). We conducted MS/MS analysis of the molecular peaks of melamine for identification purpose (Fig. 4d). We fragmented the molecular peak at an *m*/*z* of 232.96 [M + ^107^Ag]^+^, providing fragments ions at an *m*/*z* of 43.03 for [CH_3_N_2_]^+^, 68.02 for [C_2_H_2_N_3_]^+^, 148.92 for [CH_2_N_2_ + ^107^Ag]^+^, and 190.94 for [C_2_H_4_N_4_ + ^107^Ag]^+^.

Heavy demand in the food industry has led to unethical food adulteration practices with financial gains for unscrupulous producers. This adulteration resulted in the hospitalization of approximately 300,000 children in 2008 [6]. Considering the needs to track small toxins in food samples, many MS methods were developed for analysis of melamine, such as gas chromatography (GC) [52], liquid chromatography (LC) [53], and desorption electrospray-ionization (DESI) [54]. Despite reasonable progress, current MS methods normally require tedious sample pre-treatment procedures and long experimental time (~ hours). Our approach tackled the above challenges and can be employed in the food industry for large-scale application.

## Conclusion

In summary, we herein reported a plasmonic MALDI MS approach to identify and quantitate small molecules in real-case emulsion analysis. Due to the sensitive LDI process employing plasmonic silver nanoparticles as matrices, our approach afforded trace sample consumption down to ~ nL, fast analytical process in minutes, and minimum pre-treatment without enrichment/separation. This work contributes to the near-future application of MALDI MS in a broad field and personalized design of plasmonic materials for real-case bio-analysis.

## Electronic supplementary material

Below is the link to the electronic supplementary material.

Supplementary material 1 (PDF 288 kb)
