# Supplementary material for: A Plasmonic Mass Spectrometry Approach for Detection of Small Nutrients and Toxins
Source: Nanomicro Lett. 2018 May 17;10(3):52. doi: 10.1007/s40820-018-0204-6 (PMC6199099; doi:10.1007/s40820-018-0204-6)
Supplement: Supplementary file 1 — Supplementary material 1 (PDF 288 kb) [file 40820_2018_204_MOESM1_ESM.pdf]

Supporting Information for

A Plasmonic Mass Spectrometry Approach for the Detection of Small Nutrients and Toxins

Shu Wu[1,]†, Linxi Qian[2,]†, Lin Huang[1], Xuming Sun[1], Haiyang Su[1], Deepanjali D Gurav[1], Mawei Jiang[2], Wei Cai[2,]*, Kun Qian[1,]*

[1]School of Biomedical Engineering, Med-X Research Institute, Shanghai Jiao Tong University, Shanghai 200030, People's Republic of China

[2]Xinhua Hospital, Shanghai Institute for Pediatric Research, Shanghai Jiao Tong University, Shanghai 200092, People's Republic of China

†S. Wu and L. Qian contributed equally to this work.

*Corresponding authors. E-mail: k.qian@sjtu.edu.cn (Kun Qian), caiw1978@163.com (Wei Cai)

Supplementary Figures and Tables

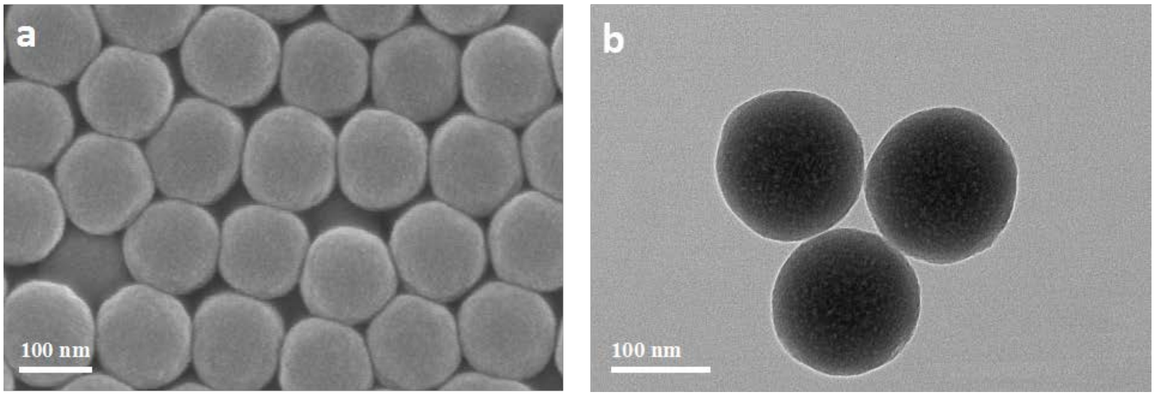

Fig. S1 Electron microscopy images of silica particles. **a** SEM and **b** TEM images of bare SiO_2 particles

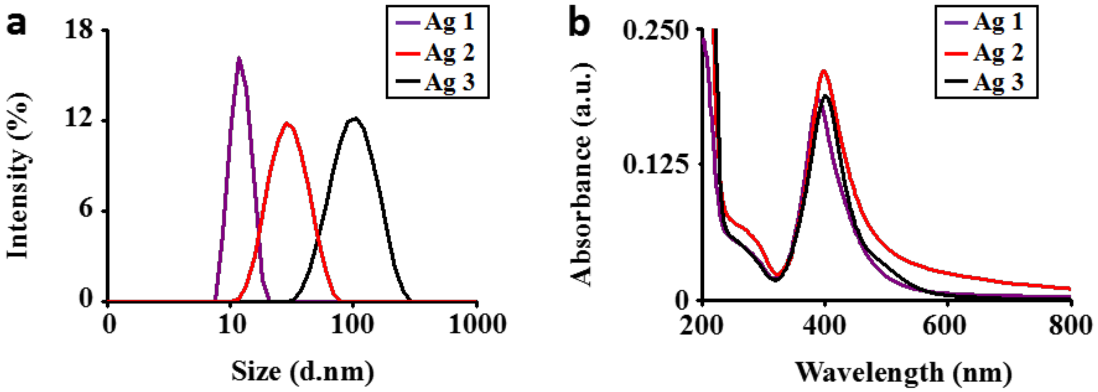

Fig. S2 a Size distribution and **b** UV-Vis absorption spectrum of Ag nanoparticles with different sizes

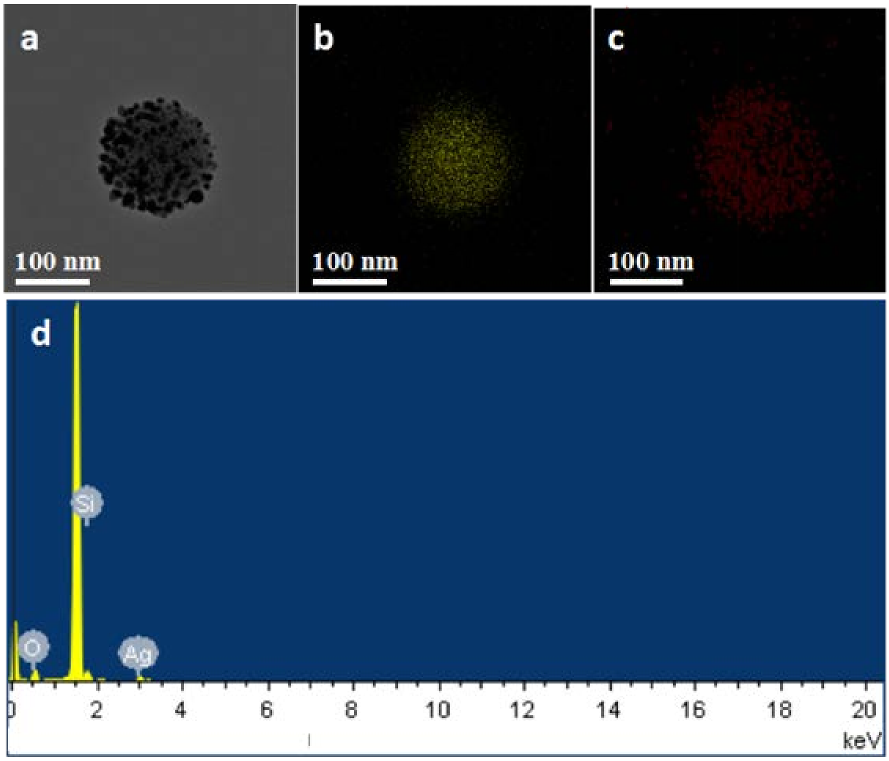

Fig. S3 Elemental mapping results. **a** STEM image, **b** silicon, and **c** silver mappings of SiO_2@Ag. **d** EDX spectra for SiO_2@Ag

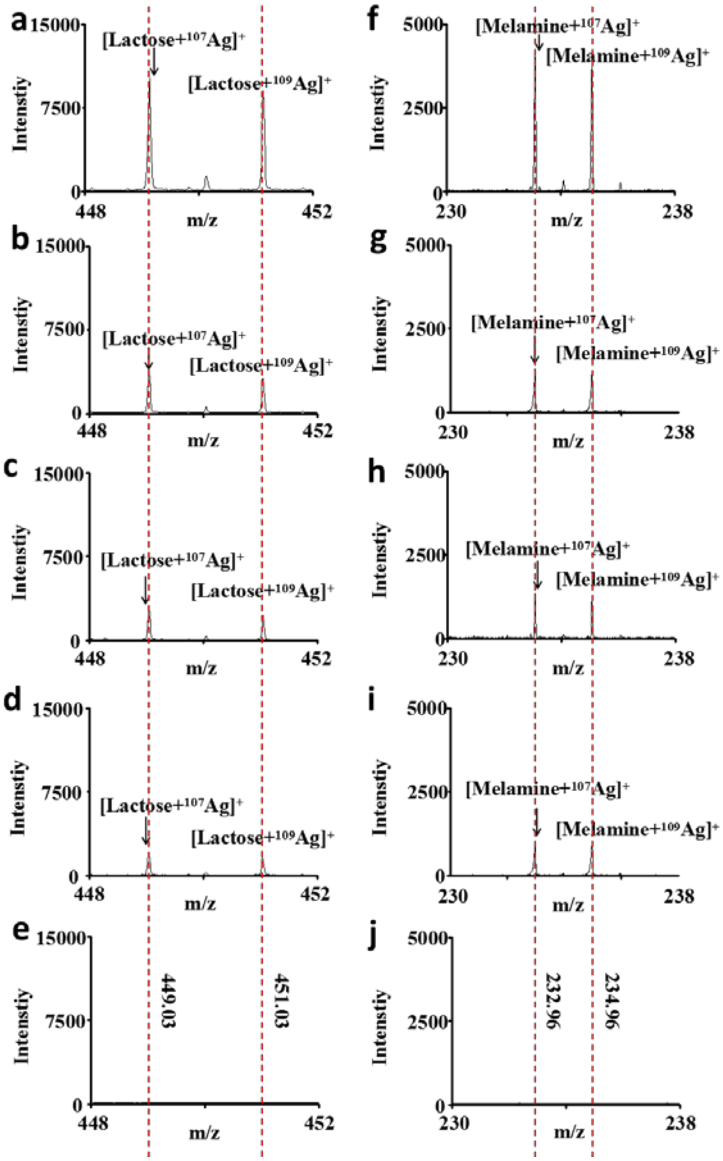

Fig. S4 LDI MS detcetion of 100 ng μL^{-1} lactose using **a** silver nanoshells, silver nanoparticles with average size of **b** 14.5 nm, **c** 34.3 nm, **d** 94.3 nm, and **e** silica spheres. LDI MS detection of 10 ng μL^{-1} melamine using **f** silver nanoshells, silver nanoparticles with average size of **g** 14.45 nm, **h** 34.3 nm, **i** 94.3 nm, and **j** silica spheres

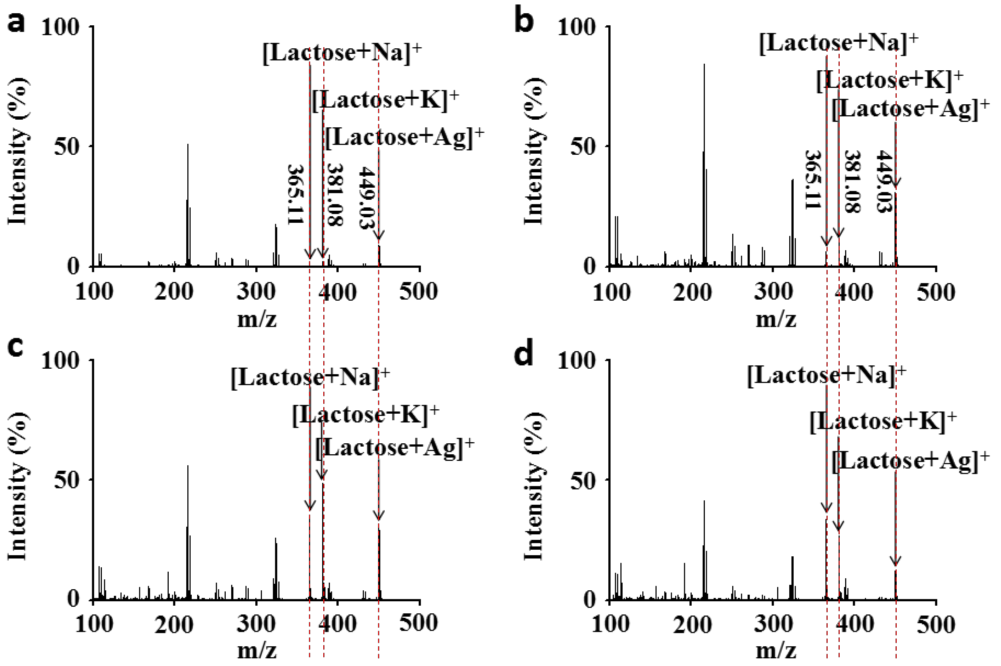

Fig. S5 Plasmonic MS analysis of lactose in dilution series of breast milk samples. **a** 50 nL with 10-fold dilution, **b** 25 nL with 20-fold dilution, **c** 12.5 nL with 40-fold dilution, and **d** 6.25 nL with 80-fold dilution. * standing for lactose

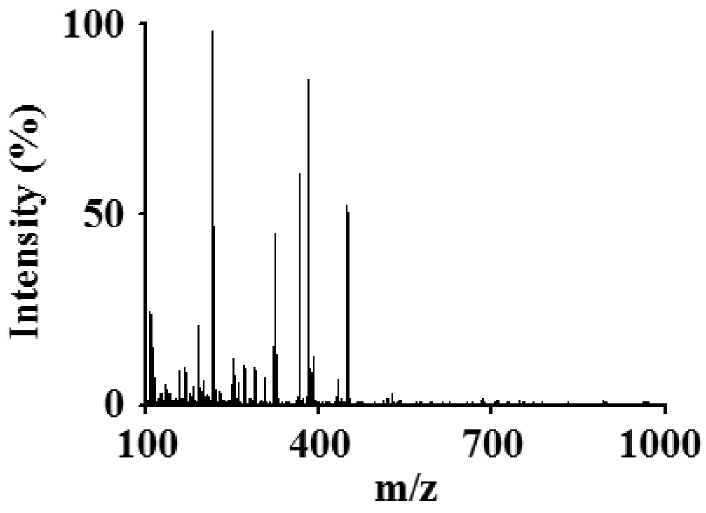

Fig. S6 Plasmonic MS analysis of breast milk samples with the m/z range of 100-1000

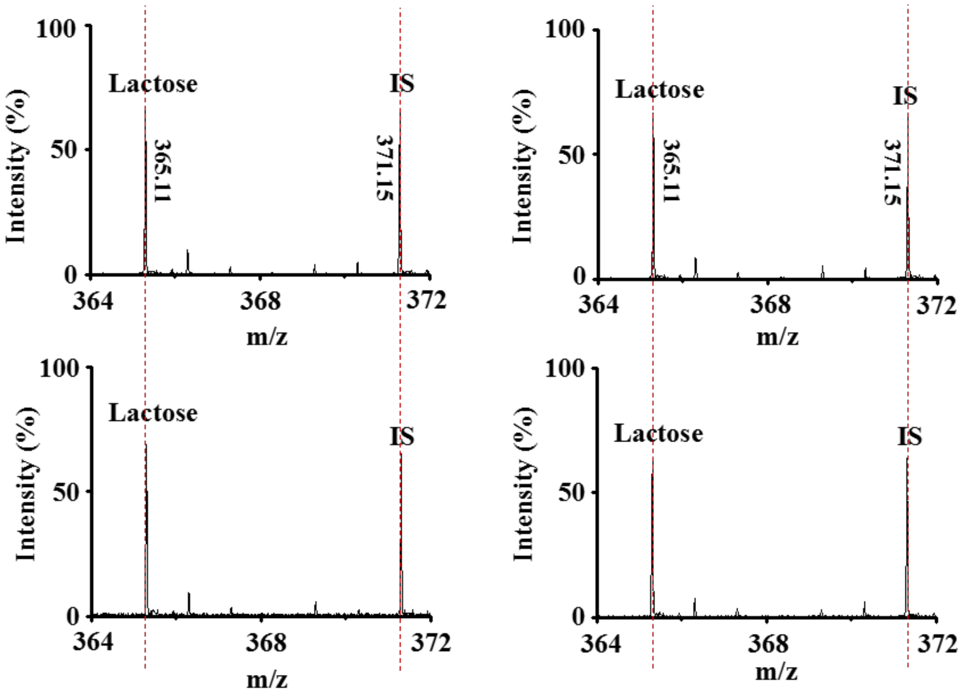

Fig. S7 Typical mass spectrum of lactose with its isotope as internal standards (IS) for quantification by the other 4 independent experiments (in addition to Fig. 3a) in parallel (A/I, 1/1).

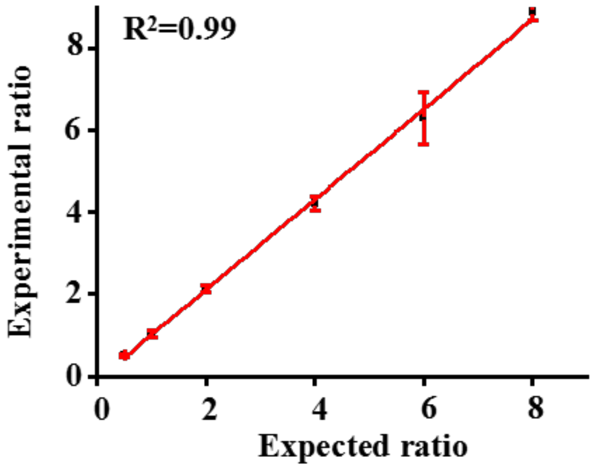

Fig. S8 The calibration curve obtained by plotting experimental ratio of analyte/isotope (A/I) for silver adducts as a function of expected ratio of A/I for lactose

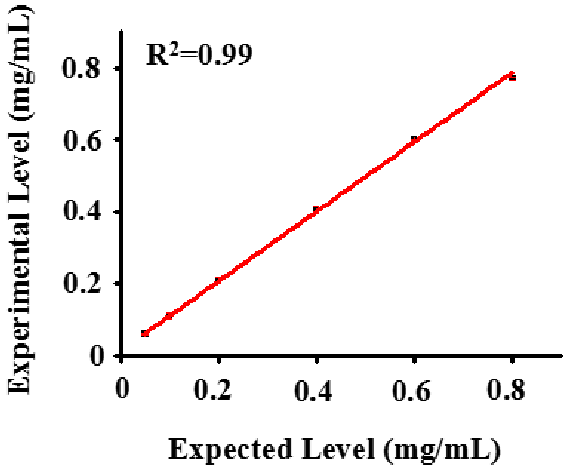

Fig. S9 The calibration curve obtained by the biochemical method. 3 independent experiments were performed for each sample to calculate the standard deviation (s.d.) as error bars. Data were shown as the mean ±s.d. (n=3)

Table S1 Structural parameters of the particles

Samples	Average Size (nm)	PDI	Zeta Potential (mV)
iO_2	167.1±1.3	0.024±0.010	-30.5±1.0
SiO_2@Ag	184.7±1.2	0.216±0.011	-21.0±0.8
Ag 1	14.5±1.2	0.191±0.020	-24.5±1.6
Ag 2	34.3±2.5	0.178±0.021	-15.3±1.9
Ag 3	94.3±7.7	0.282±0.015	-11.2±2.9

The measurements of particle size and PDI were according to the DLS experiments.

Table S2 Intraday and interday precision validation of isotopic quantification for lactose

	Average concentration (mg mL^{-1})	Intra-batch CV (%)	Batch-to-batch CV (%)
Intraday results			
40 mg mL^{-1} (n = 5)	38.88±0.77	1.93	< 5
80 mg mL^{-1} (n = 5)	79.13±1.44	1.82	< 5
Interday results			
40 mg mL^{-1} (n = 5)	39.55±0.89	2.25	< 5
80 mg mL^{-1} (n = 5)	81.18±2.49	3.07	< 5

Table S3 Lactose levels from 20 breast samples obtained by lactose assay kit and plasmonic MS

Sample number	Lactose assay kit (mg mL^{-1})	Plasmonic MS (mg mL^{-1})
1	63.09	62.75
2	63.33	60.91
3	68.70	62.57
4	72.69	69.94
5	66.55	66.60
6	64.46	62.31
7	67.51	66.81
8	67.48	67.17
9	69.60	64.45
10	69.17	67.46
11	67.57	63.27
12	59.76	56.09
13	49.14	51.93
14	59.52	56.84
15	64.40	61.75
16	66.98	61.21
17	58.20	59.97
18	60.60	58.18
19	62.61	60.58
20	67.45	62.04
